# Supplementary material for: Content-rich biological network constructed by mining PubMed abstracts
Source: BMC Bioinformatics. 2004 Oct 8;5:147. doi: 10.1186/1471-2105-5-147 (PMC528731; doi:10.1186/1471-2105-5-147)
Supplement: Additional File 5 — The original Chilibot query results of the term "long-term potentiation (LTP)" and 22 other terms, limiting the latest references analyzed to the years 1990, 1995, 2000, and 2004. [file 1471-2105-5-147-S5.bz2 › chilibotAdditionalFile5/ltp2000/html/TRKA_PKC.html]

 


 **TRKA** and **PKC** 
  
Found 9 abstracts in PubMed,  **9 abstracts were retrieved and analyzed**.  


---

 Search Google  |
 PDF files only 
|  EDU domain only 

---

**Interactive relationship** (e.g. stimulation, inhibition, etc)

**Parallel relationship** (e.g. studied together, co-existance, homology, etc.)

- A series of disubstituted alkylthio methyl and alkoxymethyl K 252a derivatives was synthesized with the aim of enhancing and separating the neurotrophic properties from the undesirable NGF trk A  [ **TRKA** ]  kinase and  **PKC**  inhibitory activities of K 252a.  Ref: 9191963 J Med Chem, 1997
- Activation of signal transduction pathways involving  **trkA** , PLCgamma 1,  **PKC**  isoforms and ERK 1 2 by tetanus toxin.  Ref: 10996319 FEBS Lett, 2000
- In the present work the differential activation of  **PKC**  isoforms and of signal transduction pathways, including nerve growth factor receptor  **trkA** , phospholipase Cgamma 1 PLCgamma 1, and extracellular regulated kinases 1 and 2 ERK 1 2 by TeTx in a synaptosome enriched P 2 fraction from rat brain is reported.  Ref: 10996319 FEBS Lett, 2000
